# Effects of Exercise on Patients Important Outcomes in Older People With Sarcopenia: An Umbrella Review of Meta-Analyses of Randomized Controlled Trials

**DOI:** 10.3389/fmed.2022.811746

**Published:** 2022-02-03

**Authors:** Yanjiao Shen, Dan Liu, Sheyu Li, Yazhou He, Fucha Tan, Xuelian Sun, Daiping Li, Xin Xia, Qiukui Hao

**Affiliations:** ^1^Department of Guideline and Rapid Recommendation, Cochrane China Centre, MAking GRADE the Irresistible Choice (MAGIC) China Centre, Chinese Evidence-Based Medicine Centre, West China Hospital, Sichuan University, Chengdu, China; ^2^Department of Endocrinology and Metabolism, West China Hospital, Sichuan University, Chengdu, China; ^3^Department of Oncology, West China School of Public Health and West China Fourth Hospital, Sichuan University, Chengdu, China; ^4^Department of Geriatrics, Affiliated Hospital of Heze Medical College, Heze, China; ^5^The Center of Gerontology and Geriatrics/National Clinical Research Center of Geriatrics, West China Hospital, Sichuan University, Chengdu, China; ^6^School of Rehabilitation Science, McMaster University, Hamilton, ON, Canada

**Keywords:** exercise, sarcopenia, meta-analyses, umbrella review, randomized controlled trials

## Abstract

**Background:**

Many clinical practice guidelines strongly recommend exercise as an intervention for patients with sarcopenia. However, the significance of exercise on patient-important outcomes in older adults with sarcopenia is inconsistent when considering available minimal important differences. To synthesize current systematic review and meta-analyses evidence on the efficacy of exercise on patient-important outcomes in the treatment of sarcopenia in older adults.

**Methods:**

We searched MEDLINE, EMBASE, Cochrane Library (Cochrane database of systematic review, CDSR) via OvidSP and Web of science until April 2021 and reference lists. Two independent investigators performed abstracted and title screening, assessed the full text and quality of evidence. This umbrella review included systematic reviews and meta-analyses of randomized controlled trials (RCTs). Eligible reviews aim to evaluate the effect of exercise on patient-important sarcopenic outcomes (muscle or physical function, mortality, and quality of life) in treating sarcopenia in older people. We used the minimally important differences (MIDs) of these outcomes to assess if the effects of exercise matter to patients.

**Results:**

This umbrella review provided a broad overview of the existing evidence and evaluated the systematic reviews' methodological quality and evidence for all these associations. In older patients with sarcopenia, moderate- to high-quality evidence showed that exercise intervention probably increases walking speed and improved physical performance (measured by TUG test); exercise may increase the muscle strength (grip strength, keen extension strength); but the effect size for grip strength probably too small to achieve patients important changes. Evidence for older people with sarcopenic obesity is limited, and we found the consistent effect of exercise interventions on grip strength and usual walking speed.

**Conclusion:**

Exercise has a positive and important effect on physical performance for older adults with sarcopenia, which supports leaving the current recommendations unchanged. New systematic reviews to summarize the effect of exercise on the quality of life are warranted to fill the current evidence gap.

## Introduction

Sarcopenia is a generalized and progressive skeletal muscle disorder that involves the accelerated loss of muscle mass and muscle function (1) and has been recognized as an independent disease with an International Classification of Diseases-10 code (M62.84) by the World Health Organization (WHO) in 2016 (2). Recognition of sarcopenia as a disease has led to major research efforts into the best screening, diagnosis, treatment, and management practices.

People with malnutrition are at a high risk of sarcopenia and these two common geriatric syndromes are closely related to each other (3). However, there is another state that co-exisit of sarcopenia and obesity, which refers to sarcopenic obesity (4). Sarcopenia synergistically worsens the adverse effects of obesity in older adults. Obesity also impairs muscle quality and decreases physical function (5, 6). Sarcopenic obesity combines the negative effects of sarcopenia and obesity in older adults and can result in metabolic problems, poor quality of life, disability, hospitalization, and death (7). By translating current, comprehensive evidence into clinical practice, it may be possible to reduce the risk for functional decline, falls, fractures, hospitalization, and mortality associated with sarcopenia or sarcopenic obesity (8, 9).

The most widely cited definition is proposed by the European Working Group on Sarcopenia in Older People (EWGSOP) (10) and updated as EWGSOP2 (11) in January 2019. In clinical practice, a person with low muscle strength and low muscle mass or quality will be diagnosed with sarcopenia by EWGSOP2. The WHO has shifted the focus of providing comprehensive care for older adults from a disease-centred model to a function-centred model. Emphasis on muscle strength and physical function can merit lifelong monitoring. According to the evidence-based clinical practice guidelines published in 2018, grip strength, keen extension strength, walking speed are regarded as critically important (12). Therefore, we defined the patient important outcome as muscle function, physical function, all-cause mortality and quality of life.

The current non-pharmacological interventions for sarcopenia are mainly exercise and nutritional interventions. Because of the lack of high-quality evidence for nutrition, in this paper, we focus on evidence examining exercise interventions compared to background therapy (with or without nutritional intervention). In detail, we included the following comparisons: exercise alone vs. usual care, exercise plus nutrition vs. nutrition alone. Based on previous systematic reviews, most guidelines provided strong recommendations for exercise/physical activity as the primary treatment for older adults with sarcopenia (12–15). A previous systematic umbrella review supports that resistance training or multimodal exercise therapy (includes a combination of resistance training, aerobic training, balance training, walking, and other types of training) can improve muscle mass, muscle strength, and physical performance in patients with sarcopenia (16). However, another umbrella review demonstrated limited quality evidence of the positive effects of mixed and resistance training in treating sarcopenia (17). Although previous systematic reviews reported that exercise had a statistically significant impact on related measurement, they did not assess if these changes exceed patients' minimal important difference (MID). Due to the inconsistency of the evidence described above and lack of considering MID among previous systematic reviews, we performed an umbrella meta-analysis based on all the current evidence already studied to understand better the role of exercise in the treatment for sarcopenia.

## Methods

### Search Strategy

We searched MEDLINE, EMBASE, Cochrane Library (Cochrane database of systematic review, CDSR) via OvidSP and Web of science until April 2021 using a comprehensive search strategy (Supplementary Table 1) to find meta-analyses or systematic review. Search terms constructed coverage for umbrella reviews according to the PICOS framework. The searches will be developed and combined using broad search terms, keywords and MeSH terms: Participants (P): sarcopenia (e.g., sarcopeni^*^ or myopeni^*^ or dynaponi^*^); Study design (S): Review, Systematic review, meta-analysis, meta-regression, meta-synthesis, realist review, realist synthesis, rapid review, pragmatic review, umbrella review. In addition, we manually searched references that were finally included in the study. This meta-analysis was conducted according to the preferred reporting items for systematic reviews and meta-analyses (PRISMA) guidelines (18).

### Selection Criteria

Two reviewers independently selected the titles, abstracts and full texts according to the inclusion and exclusion criteria. Any disagreements were solved by consensus and, if disagreement persisted, by a third reviewer. We included meta-analyses of RCTs that compared any category of exercise intervention with a control group of older patients (≥60 years) with sarcopenia. Sarcopenia diagnosed in any way was included. Also, research must be published in English. We only included the articles that have done meta-analysis, and systematic reviews without meta-analysis and animal studies were excluded.

### Data Extraction

Data were extracted by one investigator, then checked by a second investigator. We first extracted data from eligible meta-analyses on the first author, year of publication, search date, number of trials, sample size, age, gender, interventions, diagnostic criteria of sarcopenia, duration of intervention and follow-up time, metric of effect size, effect size with 95% CI and value of *I*^2^. Second, meta-analyses investigating multiple outcomes were recorded separately. Third, if there are several meta-analyses on the same intervention and the same outcome, data were extracted from the largest meta-analysis (that is, we chose the effect size of the meta-analysis with the highest number of RCTs). Among them, we included a meta-analysis of the study with the largest number of RCTs (19), in which controlled clinical trial (CCT) was also included. We excluded CCT and re-extracted and merged the data of RCTs.

### The Outcomes

The outcomes of interest were muscle or physical function, including but not limited to muscle strength, gait speed. Although sarcopenic obesity is a form of sarcopenia, it has its specific characteristics. We report the results of the study of patients with sarcopenic obesity separately. We are also concerned about the impact of exercise on mortality and quality of life in sarcopenia, as these are patient-important outcomes. We did not consider muscle mass because most people would not put much attention only on the outcome.

### Quality Assessment

We used A MeaSurement Tool to Assess systematic Reviews 2(AMSTAR2) (20) to assess the methodological quality of meta-analysis. It retains 10 of the original domains, has 16 items in total (compared with 11 in the original), has simpler response categories than the original AMSTAR (21), includes a more comprehensive user guide, and has an overall rating based on weaknesses in critical domains (20). Seven domains (item 2,4,7,9,11,13,15) can critically affect the validity of a review and its conclusions be regarded as weaknesses. AMSTAR 2 does not have an overall score. In AMSTAR 2, the methodological quality was usually categorized as high (No or one non-critical weakness), moderate (More than one non-critical weakness), low (One critical flaw with or without non-critical weaknesses), and critically low (More than one critical flaw with or without non-critical weaknesses) (20). Two authors independently assessed the AMSTAR 2; any disagreements were solved by consensus and by a third reviewer if disagreement persisted.

### Quality of Overall Evidence

We conducted quality of evidence assessment through the Grading of Recommendation Assessment, Development, and Evaluation (GRADE) framework, which evaluated the quality of evidence as high, moderate, low, and very low for each outcome in the pooled analyses (22) (see Supplementary Table 2). Two reviewers performed these assessments under the supervision of a third reviewer.

### Data Synthesis and Analysis

Instead of searching all the primary RCT studies in meta-analyses and re-analyzing the summary estimates with 95% CI, we just extracted the existing effect size and 95% CI for each outcome (23). If some meta-analyses were confounded with controlled clinical trial(CCT), we excluded the CCT data and re-combined the effect sizes. The values of *I*^2^ in related meta-analyses were extracted as the measures of heterogeneity. We would perform Egger's test to assess the publication bias when the outcomes contained at least 10 studies. We also calculated the *I*^2^ statistic to assess heterogeneity when detailed original data were available (24, 25). A P value of <0.1 for Egger's test indicated statistically significant publication bias, and values of *I*^2^ > 50% was regarded as significant heterogeneity. If the P-value of Egger's test is <0.1, it could be evidence of small study effects (24, 26, 27). To assess if the effect is important to patients in the study, we used the minimally important difference (MID) of important sarcopenic outcomes. The MID for grip strength, walking speed, and a TUG test time were 5.0 kg (grip strength) (28), 0.10 m/s (walking speed) (29), 2.1 s (TUG test time) (30), respectively.

## Results

### Literature Review

**Figure 1:**
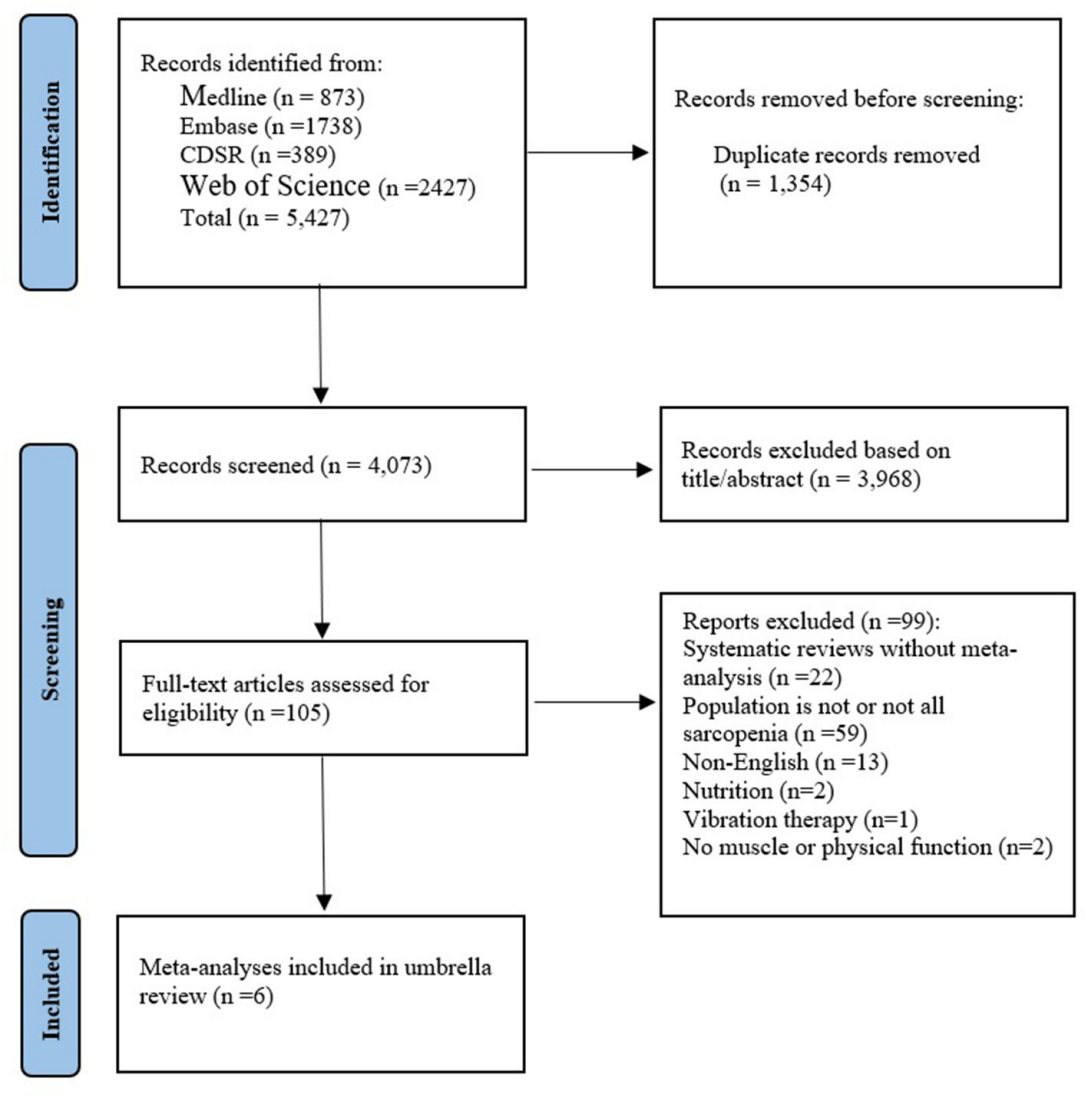
Evidence searches and selection.

As shown in Figure 1, the parallel reviews identified 4,073 unduplicated articles across 4 databases. after screening at the title and abstract level, we reviewed 105 full-text articles for eligibility. We excluded 99 articles for the following reasons: systematic reviews without meta-analysis (*n* = 22), population is not or not all sarcopenia (*n* = 57), non-English (*n* = 15), Nutrition (*n* = 2), Vibration therapy (*n* = 1), No muscle or physical function (*n* = 2). Ultimately, we included six systematic reviews and meta-analyses. Of these, three articles (15, 19, 31) had a population with sarcopenia (including 9 summary effect sizes), two (32, 33) had a population with sarcopenic obesity (including 2 summary effect sizes), and one (34) was network meta-analysis. The interventions evaluated in the meta-analyses included exercise alone vs. usual care, exercise plus nutrition vs. nutrition alone. The characteristics of the included studies were shown in Table 1.

**Table 1:** Characteristics of the included studies.

**References**	**Search date**	**No. of trials**	**Sample size**	**Age (years)**	**Gender**	**Diagnostic criteria of sarcopenia**	**Types of interventions**	**Duration (weeks)**	**Follow up duration (weeks)**
Bao et al. (19)	July-19	19	927	Ranged from 67.32 ± 5.20 and 85.90 ± 7.50	Male (143) and female (784)	ASM/Height 2, SMI, AWGS, EWGSOP, GS	Exercise programs (resistance exercise, home-based exercise, aerobic exercises, power training, whole-body vibration training and combination training)	8–36	NR
Hsu et al. (32)	April-19	14	588	Ranged from 55.0 ± 9.6 and 81.1 ± 4.6	Two studies included both men and women, two were confined to men only, 10 included only women	ASM, ASM/body weight (BW), ASM, ASM/BMI, TSM/Ht2, TSM/BW, FFM, GS	Exercise (aerobic exercise, combined exercise, power training, resistance exercise), exercise plus nutrition	8–24	12–36
Wu et al. (34)	November-20	26	2,561	65 and older	Fourteen studies included both men and women, two were confined to men only, 10 included only women, and two studies did not provide gender information	Only muscle mass, only muscle strength, muscle mass and muscle strength or physical performance	Exercise (wholebody vibration, resistance exercise, mixed exercise, other types of exercise), exercise plus Nutrition	8–48	NR
Yin et al. (33)	September-19	12	863	Mean (rang): 72.01 ± 7.76 (41–90)	Two studies included only males, 8 studies included only females, and the remaining 2 studies included mixed populations.	Muscle quantity, gait speed, GS	Exercise (aerobic exercises, resistance exercises, and exercise machines), combined intervention and electrical acupuncture.	8–28	12–28
Yoshimura et al. (15)	January-2000 to December-2016	7	751	60 years and older	Four articles are all female, one article is all female, one article is both male and female.	ASM, knee extension strength, GS, walking speed, High body fat mass, muscle mass,	Exercise Plus Nutrition	12–24	NR
Vlietstra et al. (31)	2006 to March-2017	5	415	60 years and older	Not reported	EWGSOP	Exercise intervention	12–24	NR

*AWGS, Asian Working Group for Sarcopenia; EWGSOP, European Working Group on Sarcopenia in Older People; BMI, Body Mass Index, SMI: skeletal muscle index; SMM, Skeletal Muscle Mass; NR, not reported; ASM, appendicular skeletal mass; BW, body weight; Ht2, squared body height; TSM, total skeletal mass; FFM, fat-free mass; GS, grip strength*.

### Muscle or Physical Function Outcomes of a Population With Sarcopenia

#### Exercise Intervention vs. No Exercise

Three different meta-analyses of randomized controlled studies (RCTs) (15, 19, 31) analyzed the effects of exercise intervention (vs. no exercise) with sarcopenia on 6 outcomes (Table 2), grouped as follows: muscle strength (*n* = 2) and physical performance (*n* = 4). The exercise intervention was associated with an increase in grip strength, keen extension strength, usual/max walking speed, and decline in the time of TUG test, but was not associated with five chair stand time (Table 2). Exercise intervention increased the max walking speed and its effect size exceeding the MID threshold (MD = 0.26, 95% CI: 0.14–0.38 vs. 0.1 m/s, moderate certainty). Exercise intervention lowered the time of TUG with statistically significant differences and increased usual walking speed, and their effect sizes may exceed the MID threshold (MD = 0.09; 95% CI: 0.02–0.17 vs. 0.1 m/s for usual walking speed, low certainty and MD = −1.36; 95% CI: −2.19 to −0.53 vs. 2.1s for TUG, moderate certainty). Exercise intervention increased grip strength with statistically significant differences, but the effect size did not exceed the MID threshold (MD = 1.98; 95% CI: 1.18 to 2.78 vs. 5 kg, high certainty).

**Table 2:** Comparison of overlapping results between meta-analyses of RCTs in people with sarcopenia.

**Outcome**	**Author**	**Metric**	**No. of trials**	**Sample size**	**Effect (95% CI)**	***I*^2^ %**	***P***	**Quality**
**Muscle strength**	**Exercise intervention vs. no exercise**							
Grip strength (Kg)	Bao et al. (19)	MD	13	624	1.98 (1.18 to 2.78)	41	<0.001	High
Keen extension strength (Nm/kg)	Vlietstra et al. (31)	MD	2	118	0.14 (0.03 to 0.26)	0	0.010	High
**Physical performance**								
Usual walking speed (m/s)	Bao et al. (19)	MD	10	563	0.09 (0.02 to 0.17)	85	0.010	Low
Max walking speed (m/s)	Yoshimura et al. (15)	MD	2	260	0.26 (0.14 to 0.38)	59	<0.0001	Moderate
TUG test (s)	Bao et al. (19)	MD	6	330	−1.36 (−2.19 to −0.53)	97	0.001	Moderate
Five chair stand time (s)	Bao et al. (19)	MD	4	227	−1.92 (−3.87 to 0.04)	73	0.055	Low
**Muscle strength**	**Exercise plus intervention vs. nutrition intervention alone**							
Grip strength (Kg)	Yoshimura et al. (15)	MD	2	134	0.54 (−2.90 to 3.99)	80	0.760	Low
**Physical performance**								
Usual walking speed (m/s)	Yoshimura et al. (15)	MD	3	211	0.06 (−0.01 to 0.14)	39	0.100	Moderate
Max walking speed (m/s)	Yoshimura et al. (15)	MD	2	141	0.15 (−0.15 to 0.44)	87	0.320	Low

*TUG, timed up and go; MD, mean difference; CI, confidence interval*.

In addition, the network meta-analysis in older adults with sarcopenia included 26 studies (34). Compared with the control group (no exercise), exercise increased handgrip strength (1.12 kg, 95% CI: 0.12–2.11) and improved dynamic balance assessed by timed up-and-go test and 8-foot up-and-go test (−1.76 s, 95% CI: −2.24, −1.28).

#### Exercise Plus Nutrition Intervention vs. Nutrition Intervention Alone

One meta-analysis (15) analyzed the effects of exercise plus nutrition intervention vs. nutrition intervention alone with sarcopenia on 3 outcomes (Table 2), grouped as follows: muscle strength (*n* = 1) and physical performance (*n* = 2). Nutrition plus exercise treatment was not associated with improvement in any of these outcomes compared to exercise alone (Table 2).

### Muscle or Physical Function Outcomes of a Population With Sarcopenic Obesity

#### Exercise Intervention vs. No Exercise

Two different meta-analyses of randomized controlled studies (RCTs) (32, 33) analyzed the effects of exercise intervention (vs. no exercise) with sarcopenic obesity on 2 outcomes (Table 3), grouped as follows: muscle strength (*n* = 1), physical performance (*n* = 1). The exercise intervention was associated with an increase in grip strength and usual walking speed (Table 3). Exercise intervention increases the usual walking speed with statistically significant differences and may exceed the MID threshold (MD: 0.2; 95% CI: 0.07–0.33 vs. 0.1 m/s, moderate certainty). Exercise intervention increased the grip strength with statistically significant differences but did not exceed the MID threshold (MD: 1.70; 95% CI: 0.36–3.04 vs. 5 Kg).

**Table 3:** Comparison of overlapping results between meta-analyses of RCTs in people with obesity sarcopenia.

**Outcome**	**References**	**Metric**	**No. of trials**	**Sample size**	**Effect (95% CI)**	***I* ^2^**	***P***	
	**Exercise intervention vs. no excercise**							
**Muscle strength**								
Grip strength (Kg)	Yin et al. (33)	MD	7	314	1.70 (0.36–3.04)	59%	0.0100	Moderate
**Physical performance**								
Usual walking speed (m/s)	Hsu et al. (32)	MD	5	242	0.2 (0.07–0.33)	85%	0.0020	Moderate

*MD, mean difference; CI, confidence interval*.

#### Mortality and Quality of Life

We did not find a meta-analysis of mortality and quality of life in patients with sarcopenia but did have RCTs that assessed quality of life (35–37).

#### AMSTAR Assessment

According to the AMSTAR 2, among the six included meta-analyses, no meta-analyses had moderate methodological quality, two meta-analyses had low methodological quality, and four meta-analyses had critically low methodological quality (see Supplementary Table 3).

#### Publication Bias

Egger's regression tests showed no statistically significant publication bias for grip strength (*Z* = −0.18, *P* = 0.860) and no obvious asymmetry from the funnel plots (Supplementary Figure 1). However, for usual walking speed, we found statistically significant publication bias from Egger's regression test (*Z* = 2.52, *P* = 0.036) and obvious asymmetry from the funnel plots (Supplementary Figure 2).

## Discussion

### Principal Findings

In this umbrella review, we provided a broad overview of the existing evidence and evaluated the methodological quality of the meta-analyses and quality of evidence for all these associations. In older patients with sarcopenia, moderate to high-quality evidence showed that exercise intervention probably increases usual walking speed (max) and improved physical performance (measured by TUG test); exercise may increase the muscle strength (grip strength, keen extension strength); but the effect size for grip strength probably too small to achieve patients important changes. Evidence for older people with sarcopenic obesity is limited, and we found the consistent effect of exercise interventions on grip strength and usual walking speed.

### Comparison With Other Studies

Our research supports the recommendations from the clinical guideline by Dent et al. (12) and the umbrella systematic review (16) published in 2019 (exercise therapy can improve muscle strength and physical performance in patients with sarcopenia).

We found a statistically significant effect of exercise on grip strength, but the effect may not important to patients. It may be that the type of exercise in the studies we included was not divided into subgroups according to resistance exercise, aerobic exercise and combined exercise; if it had been resistance exercise, this value would probably have reached the MID. In addition, the MIDs were not generated from sarcopenic population as there is no MID for the sarcopenic population. The MID for grip strength in the sarcopenic population may be smaller than the MID for the people we select (American adults with recent stroke), the MID is an individualized thing, the MID only reflects the mean value of the population, which may be important for some individuals to change.

### Strengths and Limitations

Our umbrella review had several strengths. (1) It provided a systematic, comprehensive overview of the evidence from all published meta-analyses regarding the role of exercise in the prevention of sarcopenia. (2) Another advantage of our literature study is that it provides a higher level of evidence than narrative reviews, and our umbrella review considers for inclusion the highest level of evidence (meta-analyses). (3) We also evaluated the methodological quality and quality of evidence by using the AMSTAR 2-criteria. Based on these scientific quality assessments, we concluded that the quality of our articles could be supported. (4) In the study, we also applied the minimally important difference (MID) for outcomes to assess if the effects matter to patients.

Our study also had several limitations. (1) Our umbrella review is dependent on the quality of the included systematic reviews/meta-analyses. We were unable to perform further subgroup analyses of exercise. Due to the lack of available evidence, we could not determine the most appropriate type (e.g., resistance exercise, aerobic exercise) or dose (e.g., duration, frequency, number of repetitions) of exercise to treat older adults with sarcopenia. (2) We did not assess the quality of individual randomized clinical trials and only combined the part of original data from selected clinical trials for analysis. (3) We ended up with a small number of included studies. This is also reflected by the fact that none of the included studies reported on the effect of exercise on mortality, quality of life, falls, fractures, etc., in patients with sarcopenia. There were also few included studies focused on obesity sarcopenia, evidence for older people with sarcopenic obesity is limited and needs further investigation. (4) Although several working groups have recommended definitions of “sarcopenia” (10, 38, 39), there are no universally accepted diagnostic criteria for sarcopenia, and these definitions vary slightly. The studies we included did not distinguish between these diagnostic criteria and included all studies that diagnosed sarcopenia, which may lead to a high degree of heterogeneity in our study. (5) Results should be viewed with caution due to the small sample size and the critically low methodology of meta-analysis.

### Future Directions

To better guide clinicians in intervening with exercise in sarcopenia, the authors recommend that researchers apply the new operational definition of sarcopenia, using the recommended cut-points for identifying participants and measuring outcomes. Although exercise appears to improve sarcopenia in the short term, studies on long-term outcomes such as quality of life, and death are still needed. Large-scale RCT studies are needed to determine which types (e.g., resistance training, mixed training) and doses (e.g., frequency, repetitions, time) of exercise are more beneficial for older patients with sarcopenia. Exercise is a relatively low-cost and potentially low-risk treatment for sarcopenia. With the growing interest in sarcopenia, we need more and better research in this area to guide clinical practice.

## Conclusion

Exercise has a positive and important effect on physical performance (walking speed and TUG test) for older adults with sarcopenia. The effect of exercise on muscle strength may not be important for older people with sarcopenia. Our results support leaving the current recommendations about exercise for older people with sarcopenia unchanged. New systematic reviews to summarize the effect of exercise on the quality of life or new clinical trials focus on all patients-important outcomes are warranted to fill the current evidence gap.

## Data Availability Statement

The original contributions presented in the study are included in the article/Supplementary Material, further inquiries can be directed to the corresponding authors.

## Author Contributions

QH and YS: study concept and manuscript editing. QH, SL, YH, and YS: study design. DLiu, XS, XX, and DLi: data acquisition, literature screening, and data extraction. SL and QH: quality control of data and algorithms. QH, YS, and FT: data analysis and interpretation. YS: manuscript preparation. QH, SL, YH, FT, and YS: manuscript review. All authors reviewed the manuscript.

## Funding

This work was supported by grants from National Key R&D Program of China (2020YFC2005600), Subject No. (2020YFC2005605), the Project of Health and family planning commission of Sichuan Province (CGY2017-101), and the Project of Science and Technology Bureau of Sichuan Province (2020YFS0167). The sponsors did not participate in the design, methods, data collection, analysis, or preparation of this manuscript.

## Conflict of Interest

The authors declare that the research was conducted in the absence of any commercial or financial relationships that could be construed as a potential conflict of interest.

## Publisher's Note

All claims expressed in this article are solely those of the authors and do not necessarily represent those of their affiliated organizations, or those of the publisher, the editors and the reviewers. Any product that may be evaluated in this article, or claim that may be made by its manufacturer, is not guaranteed or endorsed by the publisher.

## References

[1] Cruz-Jentoft AJ Sayer AA. Sarcopenia. Lancet. (2019) 393:2636–46. 10.1016/S0140-6736(19)31138-9 31171417

[2] Anker SD Morley JE von Haehling S. Welcome to the ICD-10 code for sarcopenia. J Cachexia Sarcopenia Muscle. (2016) 7:512–4. 10.1002/jcsm.12147 27891296 PMC5114626

[3] Sieber CC. Malnutrition and sarcopenia. Aging Clin Exp Res. (2019) 31:793–8. 10.1007/s40520-019-01170-1 31148100

[4] Goisser S Kemmler W Porzel S Volkert D Sieber CC Bollheimer LC . Sarcopenic obesity and complex interventions with nutrition and exercise in community-dwelling older persons–a narrative review. Clin Interv Aging. (2015) 10:1267–82. 10.2147/CIA.S82454 26346071 PMC4531044

[5] Brady AO Straight CR Evans EM. Body composition, muscle capacity, and physical function in older adults: an integrated conceptual model. J Aging Phys Act. (2014) 22:441–52. 10.1123/JAPA.2013-0009 23945551

[6] Maffiuletti NA Jubeau M Munzinger U Bizzini M Agosti F De Col A . Differences in quadriceps muscle strength and fatigue between lean and obese subjects. Eur J Appl Physiol. (2007) 101:51–9. 10.1007/s00421-007-0471-2 17476522

[7] Stenholm S Harris TB Rantanen T Visser M Kritchevsky SB Ferrucci L. Sarcopenic obesity: definition, cause and consequences. Curr Opin Clin Nutr Metab Care. (2008) 11:693–700. 10.1097/MCO.0b013e328312c37d 18827572 PMC2633408

[8] Landi F Calvani R Cesari M Tosato M Martone AM Ortolani E . Sarcopenia: an overview on current definitions, diagnosis and treatment. Curr Protein Pept Sci. (2018) 19:633–8. 10.2174/1389203718666170607113459 28595526

[9] Pérez-Zepeda MU Sgaravatti A Dent E. Sarcopenia and post-hospital outcomes in older adults: a longitudinal study. Arch Gerontol Geriatr. (2017) 69:105–9. 10.1016/j.archger.2016.10.013 27914295

[10] Cruz-Jentoft AJ Baeyens JP Bauer JM Boirie Y Cederholm T Landi F . Sarcopenia: European consensus on definition and diagnosis: report of the european working group on sarcopenia in older people. Age Ageing. (2010) 39:412–23. 10.1093/ageing/afq034 20392703 PMC2886201

[11] Cruz-Jentoft AJ Bahat G Bauer J Boirie Y Bruyère O Cederholm T . Sarcopenia: revised European consensus on definition and diagnosis. Age Ageing. (2019) 48:601. 10.1093/ageing/afz046 31081853 PMC6593317

[12] DentE MorleyJE Cruz-JentoftAJ AraiH KritchevskySB GuralnikJ . International Clinical Practice Guidelines for Sarcopenia (ICFSR): screening, diagnosis and management. J Nutr Health Aging. (2018) 22:1148–61. 10.1007/s12603-018-1139-930498820

[13] Cruz-Jentoft AJ Landi F Schneider SM Zúñiga C Arai H Boirie Y . Prevalence of and interventions for sarcopenia in ageing adults: a systematic review. Report Int Sarcopenia Initiative (EWGSOP and IWGS) Age Ageing. (2014) 43:748–59. 10.1093/ageing/afu115 25241753 PMC4204661

[14] De Spiegeleer A Petrovic M Boeckxstaens P Van Den Noortgate N. Treating sarcopenia in clinical practice: where are we now? Acta Clin Belg. (2016) 71:197–205. 10.1080/17843286.2016.1168064 27112427

[15] Yoshimura Y Wakabayashi H Yamada M Kim H Harada A Arai H. Interventions for treating sarcopenia: a systematic review and meta-analysis of randomized controlled studies. J Am Med Direct Assoc. (2017) 18:553.e551–3.e516. 10.1016/j.jamda.2017.03.019 28549707

[16] BeckwéeD DelaereA AelbrechtS BaertV BeaudartC BruyereO . Exercise interventions for the prevention and treatment of Sarcopenia. A systematic umbrella review. J Nutr Health Aging. (2019) 23:494–502. 10.1007/s12603-019-1196-831233069

[17] Moore SA Hrisos N Errington L Rochester L Rodgers H Witham M . Exercise as a treatment for sarcopenia: an umbrella review of systematic review evidence. Physiotherapy. (2020) 107:189–201. 10.1016/j.physio.2019.08.005 32026819

[18] Moher D Liberati A Tetzlaff J Altman DG. Preferred reporting items for systematic reviews and meta-analyses: the PRISMA statement. BMJ. (2009) 339:b2535. 10.1136/bmj.b2535 19622551 PMC2714657

[19] Bao W Sun Y Zhang T Zou L Wu X Wang D . Exercise programs for muscle mass, muscle strength and physical performance in older adults with sarcopenia: a systematic review and meta-analysis. Aging Dis. (2020) 11:863–73. 10.14336/AD.2019.1012 32765951 PMC7390512

[20] Shea BJ Reeves BC Wells G Thuku M Hamel C Moran J . AMSTAR 2: a critical appraisal tool for systematic reviews that include randomised or non-randomised studies of healthcare interventions, or both. BMJ. (2017) 358:j4008. 10.1136/bmj.j4008 28935701 PMC5833365

[21] Shea BJ Grimshaw JM Wells GA Boers M Andersson N Hamel C . Development of AMSTAR: a measurement tool to assess the methodological quality of systematic reviews. BMC Med Res Methodol. (2007) 7:10. 10.1186/1471-2288-7-10 17302989 PMC1810543

[22] Guyatt GH Oxman AD Vist GE Kunz R Falck-Ytter Y Alonso-Coello P . GRADE: an emerging consensus on rating quality of evidence and strength of recommendations. BMJ. (2008) 336:924–6. 10.1136/bmj.39489.470347.AD 18436948 PMC2335261

[23] Aromataris E Fernandez R Godfrey CM Holly C Khalil H Tungpunkom P. Summarizing systematic reviews: methodological development, conduct and reporting of an umbrella review approach. Int J Evid Based Healthc. (2015) 13:132–40. 10.1097/XEB.0000000000000055 26360830

[24] Egger M Davey Smith G Schneider M Minder C. Bias in meta-analysis detected by a simple, graphical test. BMJ. (1997) 315:629–34. 10.1136/bmj.315.7109.629 9310563 PMC2127453

[25] Higgins JP Thompson SG Deeks JJ Altman DG. Measuring inconsistency in meta-analyses. BMJ. (2003) 327:557–60. 10.1136/bmj.327.7414.557 12958120 PMC192859

[26] Belbasis L Bellou V Evangelou E Ioannidis JP Tzoulaki I. Environmental risk factors and multiple sclerosis: an umbrella review of systematic reviews and meta-analyses. Lancet Neurol. (2015) 14:263–73. 10.1016/S1474-4422(14)70267-4 25662901

[27] Sterne JA Sutton AJ Ioannidis JP Terrin N Jones DR Lau J . Recommendations for examining and interpreting funnel plot asymmetry in meta-analyses of randomised controlled trials. BMJ. (2011) 343:d4002. 10.1136/bmj.d4002 21784880

[28] Bohannon RW. Minimal clinically important difference for grip strength: a systematic review. J Phys Ther Sci. (2019) 31:75–8. 10.1589/jpts.31.75 30774209 PMC6348186

[29] Bohannon RW Glenney SS. Minimal clinically important difference for change in comfortable gait speed of adults with pathology: a systematic review. J Eval Clin Pract. (2014) 20:295–300. 10.1111/jep.12158 24798823

[30] Maldaner N Sosnova M Ziga M Zeitlberger AM Bozinov O Gautschi OP . External validation of the minimum clinically important difference in the timed-up-and-Go (TUG) test after surgery for lumbar degenerative disc disease. Spine. (2021). 10.1097/BRS.0000000000004128 34033596

[31] Vlietstra L Hendrickx W Waters DL. Exercise interventions in healthy older adults with sarcopenia: a systematic review and meta-analysis. Australas J Ageing. (2018) 37:169–83. 10.1111/ajag.12521 29638028

[32] Hsu KJ Liao CD Tsai MW Chen CN. Effects of exercise and nutritional intervention on body composition, metabolic health, and physical performance in adults with sarcopenic obesity: a meta-analysis. Nutrients. (2019) 11:09. 10.3390/nu11092163 31505890 PMC6770949

[33] Yin YH Liu JYW Valimaki M. Effectiveness of non-pharmacological interventions on the management of sarcopenic obesity: a systematic review and meta-analysis. Exp Gerontol. (2020) 135:110937. 10.1016/j.exger.2020.110937 32240820

[34] Wu PY Huang KS Chen KM Chou CP Tu YK. Exercise, nutrition, and combined exercise and nutrition in older adults with Sarcopenia: a systematic review and network meta-analysis. Maturitas. (2021) 145:38–48. 10.1016/j.maturitas.2020.12.009 33541561

[35] Maruya K Asakawa Y Ishibashi H Fujita H Arai T Yamaguchi H. Effect of a simple and adherent home exercise program on the physical function of community dwelling adults sixty years of age and older with pre-sarcopenia or sarcopenia. J Phys Ther Sci. (2016) 28:3183–8. 10.1589/jpts.28.3183 27942146 PMC5140826

[36] Oh MK Yoo JI Byun H Chun SW Lim SK Jang YJ . Efficacy of combined antigravity treadmill and conventional rehabilitation after hip fracture in patients with Sarcopenia. J Gerontol Seri A-Biol Sci Med Sci. (2020) 75:e173–81. 10.1093/gerona/glaa158 32592578

[37] Sen EI Eyigor S Dikici Yagli M Ozcete ZA Aydin T Kesiktas FN . Effect of home-based exercise program on physical function and balance in older adults with sarcopenia: a multicenter randomized controlled study. J Aging Phys Act. (2021) 29:1010–7. 10.1123/japa.2020-0348 34271551

[38] Chen LK Liu LK Woo J Assantachai P Auyeung TW Bahyah KS . Sarcopenia in Asia: consensus report of the Asian Working Group for Sarcopenia. J Am Med Dir Assoc. (2014) 15:95–101. 10.1016/j.jamda.2013.11.025 24461239

[39] Fielding RA Vellas B Evans WJ Bhasin S Morley JE Newman AB . Sarcopenia: an undiagnosed condition in older adults. J Am Med Direct Assoc. (2011) 12:249–56. 10.1016/j.jamda.2011.01.003 21527165 PMC3377163

