# Supplementary material for: Effects of Exercise on Patients Important Outcomes in Older People With Sarcopenia: An Umbrella Review of Meta-Analyses of Randomized Controlled Trials
Source: Front Med (Lausanne). 2022 Feb 3;9:811746. doi: 10.3389/fmed.2022.811746 (PMC8850637; doi:10.3389/fmed.2022.811746)

Effects of exercise on outcomes of sarcopenia: an umbrella review of meta-analyses of randomized controlled trials
Appendix Tables
CONTENTS

Table S1 Search strategy

i. Ovid MEDLINE(R) and Epub Ahead of Print, In-Process, In-Data-Review & Other Non-Indexed Citations, Daily and Versions(R) <1946 to April 26, 2021>

1 exp sarcopenia/ （5282）
2 (sarcopeni* or myopeni* or dynaponi*).tw. （9377）
3 ((muscle or muscular) adj2 (atroph* or wasting* or weak* or loss* or depletion*)).tw. （44598）
4 1 or 2 or 3 （52293）
5 meta-analysis.pt. （130268）
6 exp Meta-Analysis as Topic/ （21555）
7 exp meta-analysis/ （130268）
8 meta-analysis.mp. （211954）
9 exp systematic review/ （151232）
10 exp Systematic Reviews as Topic/ （5075）
11 systematic review.mp. （212298）
12 systematic* review*.tw. （206677）
13 (metaregression or meta-regression or "meta regression").tw. （9861）
14 (metanalysis or meta-analysis or "meta analysis").tw. （171271）
15 (metasynthesis or meta-synthesis or "meta synthesis").tw. （1346）
16 ("realist review" or "realist synthesis" or "rapid review" or "pragmatic review" or "umbrella review").tw. （2243）
17 5 or 6 or 7 or 8 or 9 or 10 or 11 or 12 or 13 or 14 or 15 or 16 （342512）
18 4 and 17 (873)

ii. Embase <1974 to 2021 April 26>

1 exp sarcopenia/ (13182)

2 (sarcopeni* or myopeni* or dynaponi*).tw. (15486)

3 ((muscle or muscular) adj2 (atroph* or wasting* or weak* or loss* or depletion*)).tw. (66601)

4 1 or 2 or 3 (80137)

5 exp Meta-Analysis as Topic/ (46000)

6 exp meta-analysis/ (217531)

7 meta-analysis.mp. (320441)

8 exp systematic review/ (297311)

9 exp Systematic Reviews as Topic/ (26720)

10 systematic review.mp. (376010)

11 systematic* review*.tw. (25796)6

12 (metanalysis or meta-analysis or "meta analysis").tw. (224487)

13 (metaregression or meta-regression or "meta regression").tw. (12512)

14 (metasynthesis or meta-synthesis or "meta synthesis").tw. (1533)

15 ("realist review" or "realist synthesis" or " rapid review OR pragmatic review" or " umbrella review").tw. (1202)

16 5 or 6 or 7 or 8 or 9 or 10 or 11 or 12 or 13 or 14 or 15 (539811)

17 4 and 16(1738)

iii. EBM Reviews - Cochrane Database of Systematic Reviews <2005 to April 21, 2021>

1 (sarcopeni* or myopeni* or dynaponi*).tw. (18)

2 ((muscle or muscular) adj2 (atroph* or wasting* or weak* or loss* or depletion*)).tw. (388)

3 sarcopenia.mp. (14)

4 1 or 2 or 3 (393)

5 meta-analysis.mp. (8781)

6 systematic review.mp. (7505)

7 systematic* review*.tw. (9280)

8 (metaregression or meta-regression or "meta regression").tw. (928)

9 (metanalysis or meta-analysis or "meta analysis").tw. (8782)

10 (metasynthesis or meta-synthesis or "meta synthesis").tw. (16)

11 ("realist review" or "realist synthesis" or "rapid review" or "pragmatic review" or "umbrella review").tw. (67)

12 5 or 6 or 7 or 8 or 9 or 10 or 11 (9796)

13 4 and 12(389)

v. **Web of Science:**

1. Topic: (sarcopen* OR myopeni* OR dynaponi*) (18758)
2. Topic: ((muscle OR muscular) AND (atroph* OR wasting* OR weak* OR loss* OR depletion*)) (229793)
3. #1 OR #2 (241786)
4. Topic: (“systematic review" OR ”systematic* review*" OR metanalysis OR meta-analysis OR "meta analysis" OR metaregression OR meta-regression OR "meta regression" OR metasynthesis OR meta-synthesis OR "meta synthesis" OR "realist review" OR "realist synthesis" OR "rapid review" OR "pragmatic review" OR "umbrella review") (432740)
5. #3 AND #4 (2427)

Table S2 GRADE scale for quality assessment of evidence

Outcome	No. of RCT	Risk of bias	Inconsistency	Indirectness	Imprecision	Bublication bias	Effect (95% CI)	Quality
**1. Exercise compared to control for outcomes in people with sarcopenia**								
**Muscle strength**								
Grip strength	13	Not serious	Not serious	Not serious	Not serious	Not serious	MD 1.98 (1.18 to 2.78)	High
keen extension strength	2	Not serious	Not serious	Not serious	Not serious	Not serious	MD 0.14 (0.03 to 0.26)	High
**Physical performance**								
Usual walking speed	10	Not serious	Serious	Not serious	Not serious	Serious	MD 0.09 (0.02 to 0.17)	Low
Max walking speed	2	Not serious	Serious	Not serious	Not serious	Not serious	MD 0.26 (0.14 to 0.38)	Moderate
TUG test	6	Not serious	Serious	Not serious	Not serious	Not serious	MD -1.36 (-2.19 to -0.53)	Moderate
Five chair stand time	4	Not serious	Serious	Not serious	Serious	Not serious	MD -1.92 (-3.87 to 0.04)	Low
**2. Exercise plus nutrition compared to nutrition for outcomes in people with sarcopenia**								
**Muscle strength**								
Grip strength	2	Not serious	Serious	Not serious	Serious	Not serious	MD 0.54(CI-2.90 to 3.99)	Low
**Physical performance**								
Usual walking speed	3	Not serious	Not serious	Not serious	Serious	Not serious	MD 0.06 (-0.01 to 0.14)	Moderate
Max walking speed	2	Not serious	Serious	Not serious	Serious	Not serious	MD 0.15 (-0.15 to 0.44)	Low
**3. Exercise compared to control for outcomes in people with obesity sarcopenia**								
**Muscle strength**								
Grip strength	7	Not serious	Serious	Not serious	Not serious	Not serious	MD 1.70 (0.36 to 3.04)	Moderate
**Physical performance**								
Usual walking speed	5	Not serious	Serious	Not serious	Not serious	Not serious	MD 0.2 (0.07 to 0.33)	Moderate

Note: SMI: skeletal muscle index, SMM: Skeletal Muscle Mass, ASM: appendicular skeletal mass, TSM: Total skeletal muscle mass, TUG: time up and go, MD: mean difference, SMD: standardized mean difference

Table S3 Methodological quality assessment of included meta-analyses according to the AMSTAR 2

Author	No. of 1	No. of 2	No.of 3	No. of 4	No. of 5	No.o f 6	No. of 7	No. of 8	No. of 9	No. of 10	No. of 11	No. of 12	No. of 13	No. of 14	No. of 15	No. of 16	AMSTAR 2
**Bao W,2020**	Yes	Yes	No	Partial Yes	Yes	No	Yes	No	Yes	No	No	No	No	Yes	Yes	Yes	**Critically low**
**Hsu KJ, 2019**	Yes	No	No	Partial Yes	No	Yes	Yes	No	Partial Yes	No	No	No	No	No	Yes	Yes	**Critically low**
**Vlietstra L, 2018**	Yes	No	No	Partial Yes	Yes	Yes	Yes	Partial Yes	Yes	No	Yes	No	No	Yes	Yes	No	**Critically low**
**Wu PY, 2021]**	Yes	Yes	No	No	Yes	Yes	No	Yes	Yes	No	No	No	No	Yes	Yes	Yes	**Critically low**
**Yin YH, 2020**	Yes	Yes	No	Yes	Yes	Yes	Yes	Partial Yes	Yes	No	Yes	No	No	Yes	Yes	Yes	**Low**
**Yoshimura Y, 2017**	Yes	Yes	No	Partia Yes	Yes	Yes	Yes	Yes	Yes	No	Yes	No	No	Yes	Yes	Yes	**Low**

Note:

No. of 1. Did the research questions and inclusion criteria for the review include the components of PICO?

No. of 2. Did the report of the review contain an explicit statement that the review methods were established prior to the conduct of the review and did the report justify any significant deviations from the protocol?

No. of 3. Did the review authors explain their selection of the study designs for inclusion in the review?

No. of 4. Did the review authors use a comprehensive literature search strategy?

No. of 5. Did the review authors perform study selection in duplicate?

No. of 6. Did the review authors perform data extraction in duplicate?

No. of 7. Did the review authors provide a list of excluded studies and justify the exclusions?

No. of 8. Did the review authors describe the included studies in adequate detail?

No. of 9. Did the review authors use a satisfactory technique for assessing the risk of bias (RoB) in individual studies that were included in the review?

No. of 10. Did the review authors report on the sources of funding for the studies included in the review?

No. of 11. If meta-analysis was performed did the review authors use appropriate methods for statistical combination of results?

No. of 12. If meta-analysis was performed, did the review authors assess the potential impact of RoB in individual studies on the results of the meta-analysis or other evidence synthesis?

No. of 13. Did the review authors account for RoB in individual studies when interpreting/discussing the results of the review?

No. of 14. Did the review authors provide a satisfactory explanation for, and discussion of, any heterogeneity observed in the results of the review?
No. of 15. If they performed quantitative synthesis did the review authors carry out an adequate investigation of publication bias (small study bias) and discuss its likely impact on the results of the review?
No. of 16. Did the review authors report any potential sources of conflict of interest, including any funding they received for conducting the review?

Effects of exercise on outcomes of sarcopenia: an umbrella review of meta-analyses of randomized controlled trials

Appendix Figures

CONTENTS

Figure S1 Funnel plots for grip strength

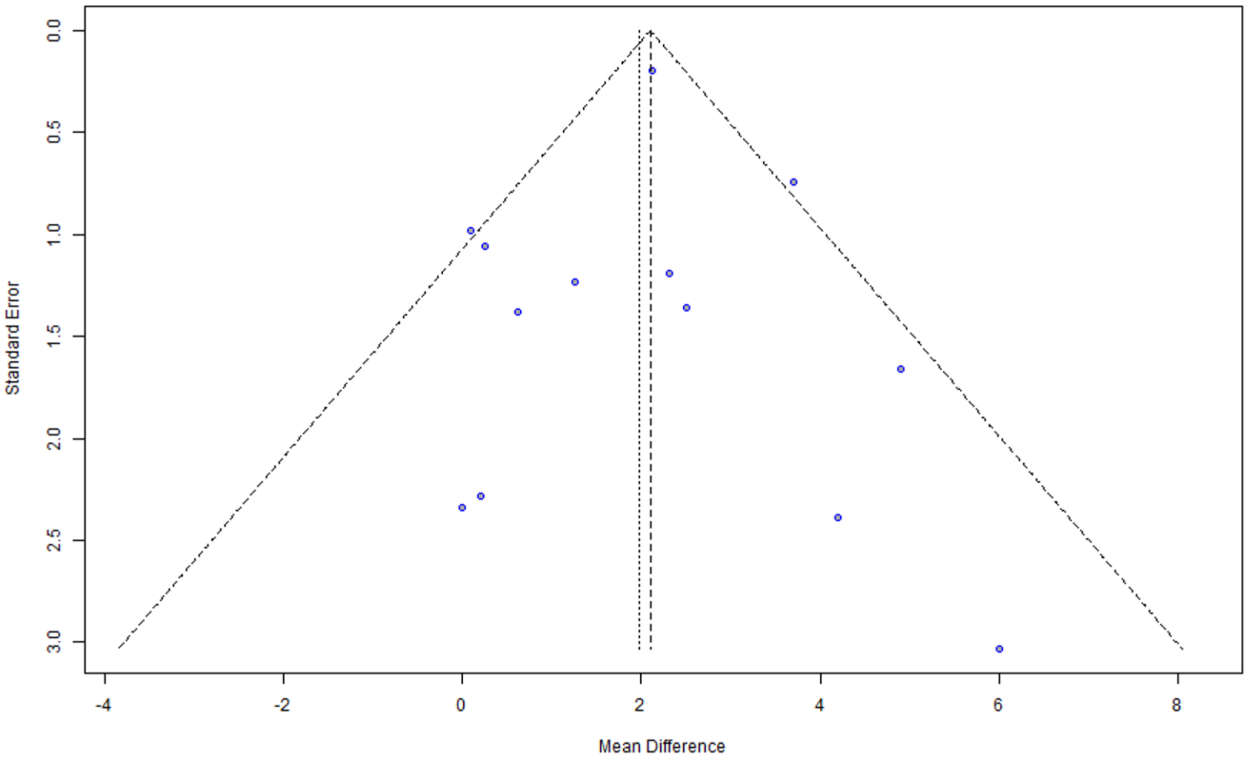

Figure S2 Funnel plots for usual walking speed

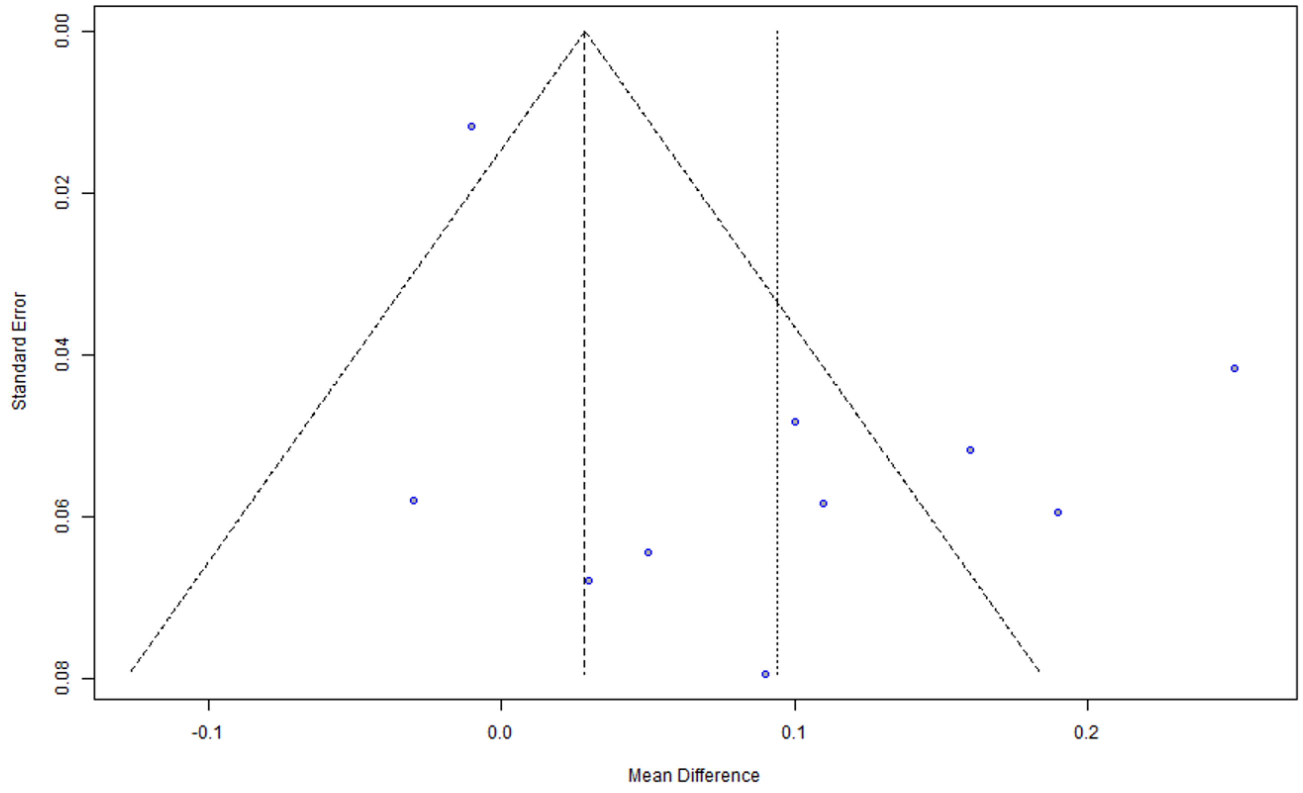

Supplement: Supplementary file 1 [file Data_Sheet_1.PDF]
